# What motivates people to defend science: Evidence from the 2017 March for science

**DOI:** 10.1371/journal.pone.0290222

**Published:** 2023-11-16

**Authors:** Eryn Campbell, John Kotcher, Teresa Myers, John Cook, Amanda C. Borth, Edward Maibach

**Affiliations:** 1 University of Pennsylvania, Penn Center for Science, Sustainability, and the Media, Philadelphia, Pennsylvania, United States of America; 2 George Mason University, Center for Climate Change Communication, Fairfax, Virginia, United States of America; 3 University of Melbourne, Melbourne Centre for Behaviour Change, Melbourne, Victoria, Australia; Yeditepe University, TURKEY

## Abstract

The 2017 March for Science was an international march organized in response to concerns over the Trump administration’s misuse of science that drew unprecedented numbers of supporters as well as attention from the media, celebrities, and political figures. The March’s turnout and publicity begs the question: what motivates people to defend science? Using data from a survey of March for Science listserv members in the US, we used a structural equation model to test posited relationships between self- and collective response efficacy, perceived threat, anger, fear, and the intention to engage in advocacy to defend science. We found that each of these constructs were associated with the intention to engage in advocacy, illuminating the motivators that lead to this intention and how individuals may be activated to engage on behalf of science in the future. These insights have both theoretical and practical significance, as advocacy is integral for both supporting and advancing fact-based policy- and decision-making.

## Introduction

Throughout history, scientists have engaged in advocacy when they felt their efforts could make a difference for society. For example, Sherwood Rowland famously called for the phasing out of chlorofluorocarbons to protect the ozone layer, and Albert Einstein notably spoke out against the rise of fascism in Germany [1,2]. Although advocacy to defend science is not necessarily a new phenomenon, events such as the formation of a political action committee to support scientists running for public office and the organization of mass demonstrations in defense of science have led some to assert that we have entered a new era of heightened political engagement by scientists and their supporters [3,4]. A notable example was the 2017 March for Science—an international event organized in response to concerns over the Trump administration’s misuse of science [3,5]. The March drew tens of thousands of participants globally and garnered substantial media coverage and the attention of celebrities and political figures, such as then President Trump [3,5]. The overwhelming participation in and publicity of the March made it a significant development in the way that scientists and the science-interested public mobilized to defend and advance shared goals through advocacy.

After the March in 2017, we conducted a survey of subscribers to the March for Science listserv to investigate the perceptions, attitudes, and motivations of people interested in the March, including scientists and non-scientists [6]. As evidence of the unprecedented mobilization it spurred, most US participants (88%) reported the March as the first science-related march they had ever participated in. This raises an important question: what motivates people to defend science? To begin answering this question, we analyzed the 2017 survey responses of people in the US, testing a series of hypotheses about the role of self- and collective response efficacy, perceived threat, anger, and fear in predicting their intention to engage in advocacy to defend science.

## Literature review

The terms advocacy and collective action have both been used to represent actions such as participating in marches or protests, voting, signing petitions, and contacting elected officials. However, there are important similarities and differences between the conceptual definitions of these constructs that guided our decision to use the term advocacy to reflect our work.

Collective action has traditionally been defined as actions that seek to improve the status or influence of a group [7,8]. More recently, collective action has been defined as, “any action that individuals undertake as group members to pursue group goals such as social change” [9, p. 122]. Further, collective action is most often used to refer to marches, protests, demonstrations, and rallies [9,10,11], although actions like signing petitions and voting have also been characterized as collective action [10].

Conversely, advocacy refers to taking action in support of a cause, idea,proposal, or policy [12–16]. Advocacy has been used to represent a wide range of actions, including but not limited to contacting elected officials, directly interacting with policymakers, writing blogs, attending public demonstrations and marches, being involved in collaborative decision-making processes, discussing an issue with others, and promoting an issue or policy via social media [16–18]. A key difference between advocacy and collective action is that advocacy does not necessarily seek to pursue the goals of a specific group, and therefore may be considered a broader term.

While the March itself may be considered an example of collective action, in this study we measured people’s intentions to engage in a diverse range of future actions—such as contacting government officials, discussing science-related issues on- and offline with friends and family, participating in a march or demonstration among others—specifically to “reduce harm to science from the current Congress and the president”. These measures better align with the conceptual definition of advocacy because they refer to a specific cause (reducing harm to science) and not about changing the status of a group directly. Therefore, we use the term advocacy when referring to our work throughout this paper. However, given their conceptual similarities, we justify our hypotheses using prior work on both advocacy and collective action, referring to the term used by the authors of these studies to accurately reflect their choice of terminology and the fields in which their research is situated. Because of this, when we cite studies to support our work, we will refer to both advocacy and collective action in an effort to accurately reflect the original choice of terminology used by the authors of these studies and the fields from which their findings are situated.

Prior research and theory suggest a range of constructs that may influence intention to engage in advocacy. To examine the relationships between several theoretically important predictors—self- and collective response efficacy, perceived threat, fear, and anger—and intentions to engage in advocacy, we drew upon two primary theoretical frameworks: the Extended Parallel Process Model (EPPM) [19,20] and the Social Identity Model of Collective Action (SIMCA) [21].

### Theoretical frameworks

Myers et al. (2018) found that the leading concerns expressed by those planning to participate in the March for Science in the US were: failure to use evidence in decision making by Congress and the Trump administration (91%); cuts to government-funded research (90%); and reduced access to government data for use in research (81%) [6]. Each of these concerns represents a unique perceived threat to the role of science in society and scientific research; several theories suggest that such threats may play an important role in peoples’ intentions to engage in advocacy.

From a communication perspective, the Extended Parallel Process Model (EPPM) is a useful theory to engage with. The EPPM explains how people respond to information about threats or risks and has been used to guide the development of health and risk communication strategies. More specifically, the EPPM posits that people’s threat and efficacy perceptions influence their responses to potentially threatening information—including attitudes, intentions, and behaviors [19,20]. Because members of the March’s listserv perceived threats to science and the role of science in governing [6], EPPM may help explain how people respond to these threats and ultimately intend to act. Other studies have drawn upon and extended the EPPM to investigate predictors of intentions to engage in advocacy to reduce climate change, such as contacting government officials, participating in a rally or protest, signing a petition, joining or volunteering with an organization, and donating money to an organization working to reduce climate change [22,23]. Here, we directly tested each of these relationships in the new context of advocacy to defend science, which expands the EPPM’s application to explain behaviors beyond those previously tested.

We also drew upon the Social Identity Model of Collective Action (SIMCA). SIMCA posits that social identity (i.e., shared social understandings of group membership) predicts collective action both directly and indirectly through perceived injustice (i.e., perceived unfair treatment or outcomes) and collective efficacy (i.e., perceived ability or effectiveness of a group to change or respond to a problem) [21,24]. Originally, van Zomeren et al. (2008) operationalized perceived injustice to encapsulate both affective (i.e., dissatisfaction, resentment, and anger) and non-affective (i.e., perceptions of unfairness, inequality, and discrimination) responses [21]. Additionally, efficacy can take different forms and therefore be operationalized in different ways [21]. We will further describe the conceptual definitions and operationalizations in this study in following sections.

Although our survey did not assess the role of identity in predicting intentions to engage in science-related advocacy, it did assess two forms of efficacy—self- and collective response efficacy—and thus connects to SIMCA. Further, drawing on the focus of negative affective responses to perceptions of injustice in SIMCA, we examined the role of anger and fear as moderators of the relationship between self- and collective response efficacy and perceived threats.

**Fig 1:**
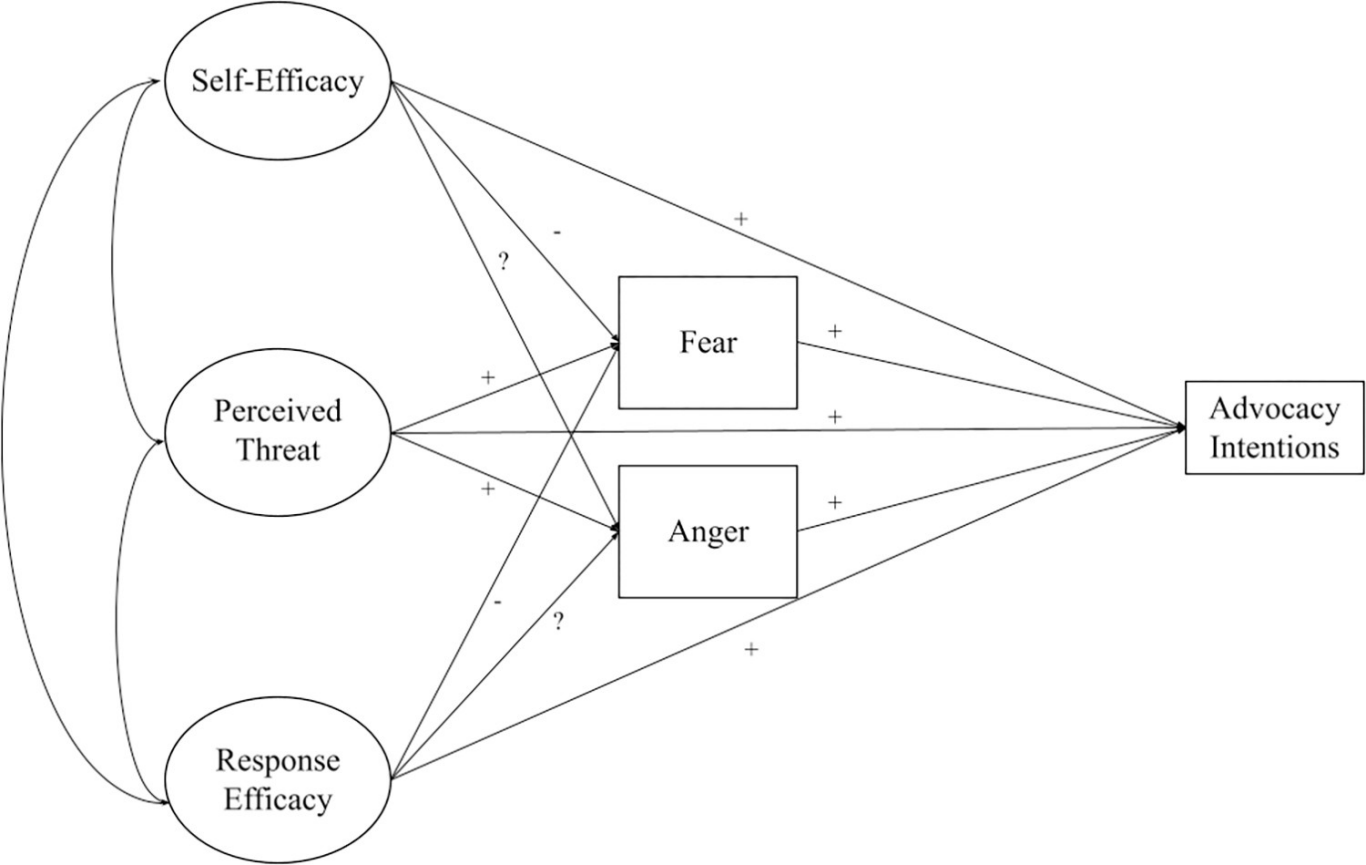
Hypothesized model of advocacy intentions.

While other theories are relevant to intentions to engage in advocacy, we drew primarily on the EPPM and SIMCA given their direct applicability to our hypotheses about predictors of future intentions to engage in science-related advocacy and about the relationships among those predictors. In the following sections, we examine each of the predictors included in our model and how theory suggests they may be related to intentions to engage in advocacy for science (Fig 1).

### Efficacy

This study is principally interested in two types of efficacy—self- and collective response efficacy—as they may have nuanced relationships with intentions to engage in advocacy to defend science. Self-efficacy refers to people’s beliefs about their ability to take specific actions [25]. Response efficacy—which Bandura (1986) called outcome expectancy—refers to people’s beliefs about the effectiveness of a specific action in a response to a threat [19,22,23,25,26]. Drawing on the EPPM, the type of actions people take in response to a threat will depend greatly on both their self- and response efficacy [19,20]. In support of this, Hart and Feldman (2016) found that self- and response efficacy all lead to intentions to engage in advocacy to reduce climate change, which included signing petitions, contacting government officials, and participating in rallies [23]. However, it is important to note that Hart and Feldman (2016) investigated what they termed as internal, external, and response efficacy. They state that internal efficacy captures the “ease with which an individual can take action in the political sphere” [23, p. 4] and is conceptually similar to self-efficacy as we define it here. Further, their definition of response efficacy aligns directly with how we define it in this study. Based on this prior work, and because the March was held in response to a threat, the relationships of these constructs with intentions to engage in advocacy to defend science is an extension of the EPPM that has not yet been explored.

Reinforcing the potential importance of efficacy, studies that employed the SIMCA theoretical framework have shown that different types of efficacy can be an influential antecedent of intentions to engage in collective action. Some studies drawing on SIMCA have investigated the role of individual efficacy or “beliefs that individuals can achieve individual goals through individual effort” and found that it was not related to intentions to sign petitions or participate in demonstrations [27, p. 621]. However, this is conceptually different from how we define self-efficacy, as it is focused on people believing they can achieve an individual goal through action rather than their ability to perform the action. Therefore, we suspected that self-efficacy as we define it may still be important for driving intentions to engage in advocacy.

More aligned with the current study, SIMCA studies have also looked at participatory efficacy or the belief that one’s engagement in collective action will contribute to making a difference. Based on this definition, participatory efficacy is conceptually similar to response efficacy. Studies have found that it has a unique influence compared to other forms of efficacy on intentions to take collective actions, like signing petitions and participating in demonstrations [27]. Further, participatory efficacy has also been shown to be positively related to intentions to engage in protests [28]. Based on this prior work, we hypothesize about both self- and response efficacy:

H1: Self-efficacy will have a positive direct effect on advocacy intentions.
H2: Response efficacy will have a positive direct effect on advocacy intentions.

### Perceived threat

A threat is a known or unknown danger or harm in an environment [19]. Studies have examined the relationship between perceived threats and advocacy intentions. For example, one study found that perceived threats were positively related to intentions to engage in collective action, which included actions like signing petitions, discussing issues on social networks, and participating in demonstrations [29]. Another study investigating support for collective action in the context of conflicts between the Turkish and Kurdish people found that perceived threats associated with these conflicts led to increased willingness to sign a petition or participate in a peaceful demonstration [30].

The March was a response to a perceived threat to science by the Trump Administration. Almost immediately after the Trump administration gained power, warning bells began sounding in the scientific community and beyond, including concern about scientific budget cuts [31], scientific censorship [32,33] and removal of scientific information from the policy making process [34]. The EPPM posits that perceptions of the threat motivate action, not the threat itself [19]. Following this, a perceived threat to science (like those that inspired the March) may lead to increased intentions to engage in advocacy. Further, it is possible that those on the March listserv may have viewed threats to science as a form of injustice, potentially aligning with the relationship put forth in SIMCA [21]. Here, our conceptualization of a perceived threat to science aligns with those posed by the EPPM and SIMCA. Therefore, we hypothesize:

H3: Perceived threat will have a positive direct effect on advocacy intentions.

### Fear

Fear is evoked when a threat is perceived, and the situation seems outside of one’s control [35–37]. In the case of threats against science, higher levels of perceived threat to science may lead to higher levels of fear. Further, the EPPM posits that efficacy messages minimize fear by allowing people to face the source of the fear, motivating them to take protective actions for their health [19,20]. As such, higher levels of self-efficacy and response efficacy may help limit the amount of fear felt by an individual. However, one study found nuanced relationships between response efficacy and fear in the context of climate action, such that response efficacy increased fear among conservatives while it decreased fear among moderates and liberals [22]. Given that we are looking at advocacy intentions amongst those who signed up to be on the March’s listserv in this study and therefore have implicitly signaled some form of wanting to engage, we hypothesize that self- and response efficacy will function to limit people’s fear while perceived threat functions to increase it:

H4: Self-efficacy will be negatively related to fear.
H5: Response efficacy will be negatively related to fear.
H6: Perceived threat will be positively related to fear.

One study found that fear has a negative relationship with political campaign participation and a positive relationship with non-voting behaviors (e.g., talking to others about voting, wearing a campaign button, attending a rally, working for a campaign, or donating money) [38]. However, fear has also been shown to have a positive relationship with intentions to engage in advocacy. For example, one study found that fear had a positive relationship with advocacy to reduce climate change, such as participating in a rally or protest or signing petitions [22]. Similarly, another study found that fear was positively related to environmental action intentions, which included actions such as doing something with others to fight climate change, signing petitions, and voting for a climate-friendly political party [39]. These discrepancies demonstrate the complex role of emotions in motivating (or depressing) political engagement. Because this study focuses on intentions to engage in advocacy to defend science—not strictly political campaign participation—we posited that a positive relationship between fear and these intentions would be more likely to be present. Therefore, we hypothesize:

H7: Fear will be positively related to advocacy intentions.

### Anger

Unlike the other variables in our hypothesized model, anger is not a construct in the EPPM; however, SIMCA has established the role of negative emotions and their importance to collective action behaviors. In SIMCA, van Zomeren et al. (2008) predicted that negative emotions (including anger) stemming from a perceived injustice (i.e., perceptions of unfair treatment or outcomes) toward one’s in-group is a central determinant of participation in collective action [21]. Here, we investigate anger’s relationship with perceived threats to science, which many on the March’s listserv may think of as a form of injustice; this perception may lead to a positive relationship between threat perceptions and anger. Additionally, research on judgment and choice has shown that perceived threats can trigger anger depending on individual perceptions of the cause of the threat and their level of control over it [35,36,40]. Therefore, we hypothesize:

H8: Perceived threat will be positively related to anger.

Less is known about the relationship between forms of efficacy and anger, although some studies have begun to explore this possibility. Valentino et al. (2009) demonstrated that efficacy may be positively related to anger towards policy threats under certain conditions [38]. Other studies have found a negative relationship between self-efficacy and anger [41]. Further, one study investigated the influence of efficacy messages on emotions and found that messages that highlight the response efficacy of climate actions by the EPA did not influence anger [22]. More research is needed to fully understand the relationships between forms of efficacy and anger. As such, both self- and response-efficacy are included as antecedents to anger in our model, but to more deeply understand how these constructs are related, we ask:

RQ1: Will self-efficacy and anger be related to one another?
RQ2: Will response efficacy and anger be related to one another?

SIMCA posits that anger from perceived injustices (and other negative emotions) directly lead to increased intentions to engage in collective action [21]. One study that drew upon the SIMCA framework demonstrated that anger was positively related to intentions to participate in protests [42]. Beyond SIMCA, another study demonstrated that anger positively predicted both an individual measure of campaign participation and a combined measure of non-voting behaviors that included the actions of talking to others about voting, wearing a campaign button, attending a rally, working for a campaign, and donating money [38]. Further, another study demonstrated that anger consensus messaging can enhance support for climate action and mobilize action intentions [43]. Therefore, we hypothesize:

H9: Anger will be positively related to advocacy intentions.

## Methods

### Participants

This study was approved by George Mason University’s Institutional Review Board (IRB number: 1055011–4) and written consent was received from all participants. Between June 8, 2017 and July 10, 2017, we fielded a survey to people subscribed to the March for Science listserv. The March for Science organization emailed a link to the online Qualtrics survey to their listserv. At the time of the survey, the listserv consisted of approximately 213,000 individuals. A total of 20,808 individuals consented to and completed at least a portion of the survey, a completion rate of 9.7%.

To investigate only those who participated in the US, the dataset was subset based on a question in the survey that asked: “Where did you participate in the March?”, with response options of (1) D.C. (*n* = 5135), (2) elsewhere in the US (*n* = 10,082), (3) outside of the US (*n* = 651). Only those who chose responses 1 or 2 were included in the analyses. Further, we asked survey participants about their participation in the March for Science as well as their perceptions of the state of science. All participants were presented a core set of questions and then randomly assigned to one of three different survey paths (enabling us to assess more topics of interest): (1) perceptions of threat to science and efficacy in addressing the threats, (2) the frequency and type of actions people engaged in, and (3) perception of the role of scientists in the public sphere. Only US participants in the threat/efficacy path (1) were included in the analysis (*N* = 4442), as this path included the questions representing the constructs of interest. Table 1 provides descriptive statistics about the included participants.

**Table 1:** Participant sociodemographic information.

Category	*n*	*%*
Gender		
Male	1456	32.8
Female	2743	61.8
Age (in years)		
18–29	686	15.4
30–49	1401	31.5
50–64	1335	30.1
65+	777	17.5
Race		
White	4176	94.0
Non-white	64	1.4
Education		
No formal education	7	0.1
High school	51	1.1
Some college, no degree, Associate’s degree	372	8.4
Bachelor’s degree	1122	25.3
Advanced degree	2734	61.5
Profession		
Scientist	1134	25.5
Engineer	298	6.7
Student	350	7.9
Medical Professional	343	7.7
Science Teacher	271	6.1
Not a scientist, but work at an organization or company that focuses on science	255	5.7
Does not work in science, but cares about it	1094	24.6
Other	694	15.6
Participation in the March		
Yes, in person	4102	92.3
Yes, digitally	340	7.7
No	0	0
Participation Location		
Washington D.C.	1552	34.9
Elsewhere in the US	2890	65.1

*Note*. *N* = 4442.

### Measures

#### Self-efficacy

We drew inspiration from Doherty and Webler (2016) for our measure of self-efficacy [26]. Participants were asked to indicate their level of confidence in their ability to engage in seven actions to reduce harm to science from Congress and the president (in 2017). The seven actions were: contacting government officials (SE1), donating money to a scientific or political organization (SE2), attending a march or public demonstration (SE3), discussing science-related issues online (e.g., blog, Facebook) (SE4), discussing science-related issues with friends and family (offline) (SE5), engaging with the media (e.g., letter to the editor, talking with journalists, radio call-in show) (SE6), and giving a non-technical public talk about their research (only asked of scientists) (SE7). Response options ranged from 1 = Not at all confident to 5 = Extremely confident. SE7 was also not used in the analysis because it was only asked of scientists.

#### Collective response efficacy

Like our measure of self-efficacy, we again used Doherty and Webler (2016) as a model for our measure of collective response efficacy [26]. Participants were asked to indicate how effective seven actions would be at reducing harm to science from Congress and the president (2017) if “many other people who share your views do it.” The seven actions were: contacting government officials (RE1), donating money to a scientific or political organization (RE2), attending a march or public demonstration (RE3), discussing science-related issues online (e.g., blog, Facebook) (RE4), discussing science-related issues with friends and family (offline) (RE5), engaging with the media (e.g., letter to the editor, talking with journalists, radio call-in show) (RE6), and giving a non-technical public talk about their research (only asked of scientists) (RE7). Response options ranged from 1 = Not at all confident to 5 = Extremely confident. RE7 was omitted from the analyses because it was only asked of scientists.

#### Perceived threat

In a matrix of seven items, participants were asked “To what extent (if at all) do you think the current Congress and the president will harm the following aspects of science…” The seven items were: the use of scientific evidence in government decision-making (PT1), the ability of scientists to freely conduct their research (PT2), the ability of scientists to freely communicate their research (PT3), the ability to access government data for scientific research (PT4), the government’s funding for scientific research (PT5), the nation’s ability to expand and diversify the scientific workforce (PT6), and the quality of science education (PT7). Response options ranged from 1 = Not at all harmful to 5 = Extremely harmful.

**Fear.** Participants were asked, “To what extent do you feel the following emotions about the potential for harm to science from the current Congress and president? I feel: Fear.” Response options ranged from 1 = Not at all to 5 = Extremely.

#### Anger

Participants were asked, “To what extent do you feel the following emotions about the potential for harm to science from the current Congress and president? I feel: Angry.” Response options ranged from 1 = Not at all to 5 = Extremely.

#### Advocacy intentions

We drew inspiration from previous work by van Zomeren et al. (2008) and Besley et al. (2013) to design our measure of advocacy intentions [21,44]. Participants were asked, “Over the next 6 months, how likely are you to engage in the following actions to reduce harm to science from the current Congress and the president?” The seven actions were: contacting government officials (AI1), donating money to a scientific or political organization (AI2), attending a march or public demonstration (AI3), discussing science-related issues online (e.g., blog, Facebook) (AI4), discussing science-related issues with friends and family (offline) (AI5), engaging with the media (e.g., letter to the editor, talking with journalists, radio call-in show) (AI6), and giving a non-technical public talk about their research (only asked of scientists) (AI7). Response options ranged from 1 = Not at all likely to 5 = Extremely likely. Based on findings of the measurement model (see Table 2), AI2 was ultimately removed from the analysis to keep consistency among the measures, as the response options and wording were identical between the efficacy items and the advocacy intentions items. AI7 was omitted from the analyses, as it was also only asked of scientists. The remaining five items were averaged to calculate a score for overall intention to engage in advocacy (α = .62). Because we were interested in predicting whether or not people engaged in any of these forms of advocacy, this was treated as an observed variable in the analyses.

**Table 2:** Summary of measurement items, single indicators, and factor loadings on measurement models.

Variable	*M*	*SD*	Factor loadings		
			Model 1	Model 2	Final Model
*Self-efficacy*					
SE1	3.34	1.26	.566	.534	.535
SE2	3.36	1.26	.361	-	-
SE3	3.77	1.06	.658	.621	.622
SE4	3.41	1.34	.626	.657	.656
SE5	4.11	0.97	.691	.712	.712
SE6	2.60	1.26	.524	.534	.533
*Collective response efficacy*					
RE1	3.36	1.09	.610	.560	.560
RE2	3.42	0.96	.604	-	-
RE3	3.09	0.99	.757	.730	.730
RE4	2.79	1.12	.789	.824	.824
RE5	3.10	1.11	.753	.780	.780
RE6	3.23	1.02	.730	.725	.725
*Perceived threat*					
PT1	4.50	0.78	.595	.595	.619
PT2	4.11	0.95	.848	.849	.773
PT3	3.97	1.09	.814	.815	.731
PT4	4.25	0.96	.714	.712	.735
PT5	4.50	0.78	.595	.597	.645
PT6	4.10	1.03	.619	.621	.662
*Fear*	3.60	1.20			
*Anger*	4.41	0.91			
*Advocacy Intention*	3.29	0.78			

*Note*. Variables appear in the same order as presented in the methods section for reference.

#### Sociodemographics

Gender was coded as 0 = Male and 1 = Female. Age was included as a continuous variable. Race was coded as 0 = White and 1 = Non-white. Education was coded on a 5-point scale, with 1 = No formal education and 5 = Advanced degree.

### Analysis

To test the hypothesized model, a structural equation model (SEM) with maximum likelihood estimation was conducted using the *lavaan* package in R [45,46]. SEM controls for measurement error and estimates model fit [47]. A two-step analytic approach was conducted [47,48]. First, a measurement model was estimated using confirmatory factor analysis (CFA) to verify the measurement components of the latent variables. Second, a partially latent structural equation model was used to test the hypothesized relationships between the latent and observed variables. The sociodemographic variables of gender, age, race, and education were included as controls in all paths of the partially latent structural equation model.

The analysis was conducted to examine how well the hypothesized model fit the data based on the following fit indices and their thresholds for good model fit: the maximum likelihood chi-square (*p* > .05), Comparative Fit Index (CFI > .95), the Root Mean Square Approximation (RMSEA < .06) and its 90% upper confidence interval (UCI < .10), and the Standardized Root Mean Square Residual (SRMR < .08) [47].

## Results

### Measurement model

First, we ran a measurement model, with three latent variables specified: self-efficacy, perceived threat, and collective response efficacy. Means, standard deviations, and factor loadings for the measurement models can be found in Table 2. Correlations between all variables in the model can be found in the S1 Table in S1 File. Because the response items for self- and collective response efficacy were identical with only the question stems varying, the error variances for all items on self-efficacy were allowed to correlate with the matching items on collective response efficacy in the model.

The model fit statistics for the first measurement model did not reach the standard thresholds (Table 3). Based on the low factor loading of SE2, the decision was made to remove this item from the analyses. Further, because of the identical wordings of the items included in the self-efficacy, collective response efficacy, and intentions to engage in advocacy measures, RE2 and AI2 were also removed from the analyses to keep the items included consistent across the measures.

**Table 3:** Measurement and structural model fit indices.

Model	*χ* ^2^	*df*	CFI	SRMR	RMSEA (90% CIs)
*Measurement Models*					
Model 1	2411.74***	126	.916	.042	.066 (.064, .069)
Model 2	1504.97***	96	.942	.036	.060 (.057, .062)
Final Model	1215.55***	95	.954	.033	.053 (.051, .056)
*Structural Model*					
Model 1	2175.60***	186	.923	.035	.055 (.053, .056)
Final Model	2018.23***	185	.929	.034	.053 (.051, .055)

Note.

*p < .05

**p < .01

***p < .001.

A new measurement model was specified after the removal of SE2 and RE2. However, the model fit statistics still did not reach the standard thresholds (Table 3). Based on the modification indices and because conducting and communicating research are closely related conceptually, the error variances between the ability of scientists to freely conduct their research (PT2) and the ability of scientists to freely communicate their research (PT3) were allowed to correlate in the model. This modification significantly improved the model, *Δχ*^2^(1) = 289.41, *p* < .001.

The model fit statistics for the third and final measurement model met the standard thresholds, except for the significant chi-square value; however, as this test is inflated by large sample sizes, we chose to accept this second measurement model as sufficient (Table 3) [47]. For parsimony, we chose not to make any more modifications, including those that the modification indices suggested the model may be improved. All factor loadings in the final measurement model were above .50, demonstrating acceptable convergent validity (i.e., the items measure the latent variable) (Table 2). Covariances between the latent variables were low (all < .35), indicating acceptable divergent validity (i.e., the latent variables are distinct). Several large residuals were present in the standardized residual matrix. These residuals may be caused by the similarity of items within and across the measures for self-efficacy and collective response efficacy. To maintain parsimony and due to the presence of good model fit statistics and acceptable factor loadings, we proceeded to run the structural model despite these remaining large residuals.

### Structural model

Next, we added the directional paths and observed variables to fit a structural model. Because fear and anger are both discrete negative emotions that were measured within the same question block in the survey, the error variances were correlated between the emotional responses of fear and anger. The model fit statistics of the hypothesized, partially latent, SEM model did not reach standard thresholds (Table 3). Therefore, we assessed the modification indices from the model, which indicated that correlating the error variances between discussing science-related issues online (e.g., blog, Facebook) (RE4) and discussing science-related issues with friends and family (offline) (RE5) would improve the model. Because the item wording is closely related, this modification was added and significantly improved the model, *Δχ*^2^(1) = 157.37, *p* < .001.

**Fig 2:**
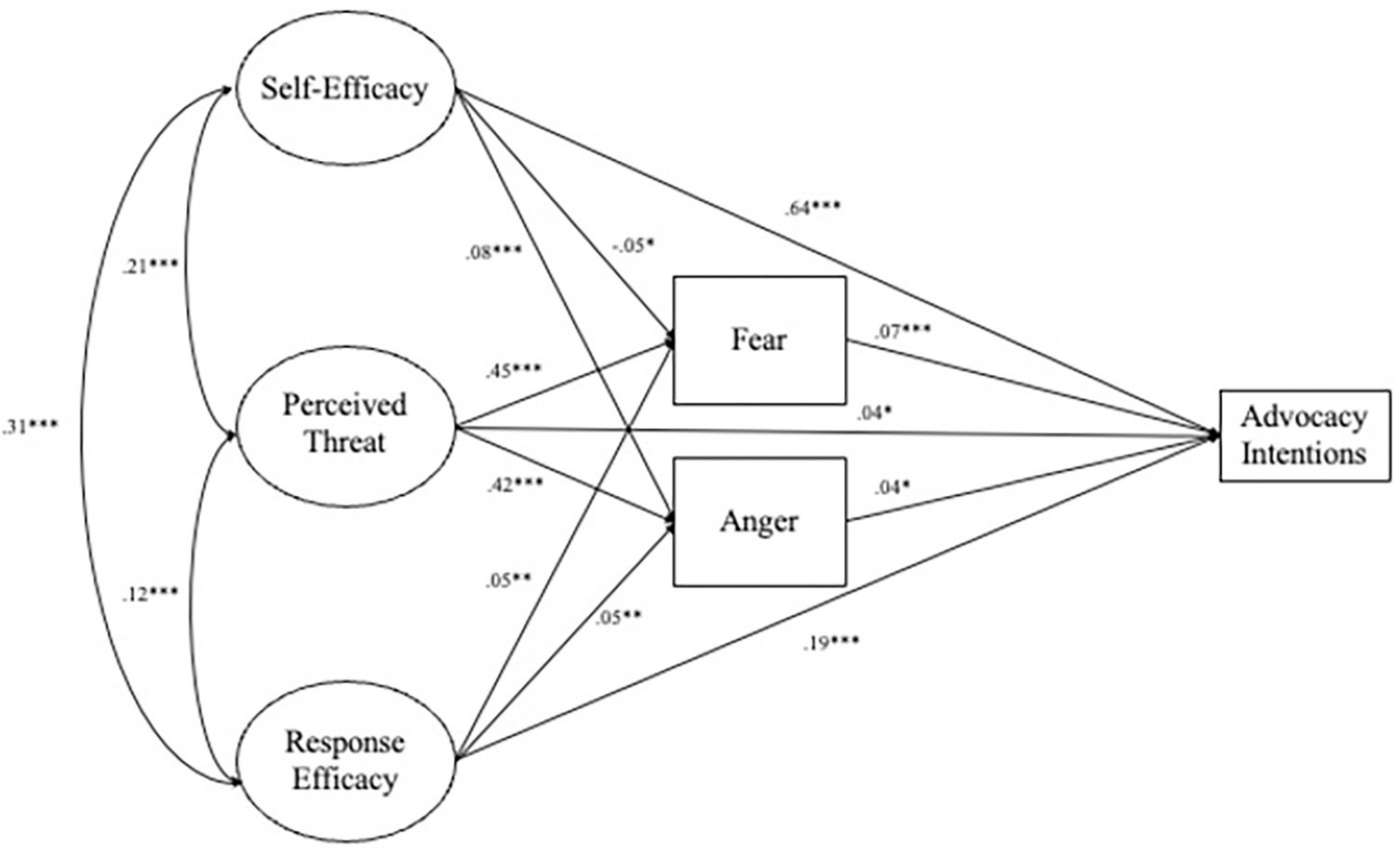
Final structural equation model with standardized path coefficients. The covariance between fear and anger is not depicted for readability (cov_Fear-Anger_ = .23***). The covariance between RE4 and RE5 is not depicted as it occurs within the latent structure of collective response efficacy (cov_RE4-RE5_ = .29***). *p < .05, **p < .01, ***p < .001.

With these modifications, the resulting model fit indices did not reach all standard thresholds; however, we chose not to further modify the model for parsimony and because the SRMR and RMSEA values remained within standard thresholds throughout the analyses (Table 3). Like in the measurement model, several large residuals were still present in the standardized residual matrix. This may be in part due to advocacy intentions having the same response options as self- and response-efficacy. Again, based on the model fit indices and for parsimony, no further adjustments were made to the model. The results of the final model are shown in Fig 2.

Self- and collective response efficacy, and perceived threat accounted for 23.3% and 21.2% of the variance in fear and anger, respectively. Collectively, self- and collective response efficacy, perceived threat, fear, and anger accounted for 57.1% of the variance in the intention to engage in advocacy. Direct, indirect, and total effects can be found in Table 4.

**Table 4:** Direct, indirect, and total effects.

Paths	*β*	*SE*
*Direct*		
Threat → fear	.045***	.055
Threat → anger	.416***	.040
Threat → advocacy intentions	.044*	.031
SE → fear	-.048*	.033
SE → anger	.080***	.025
SE → advocacy intentions	.639***	.027
RE → fear	.051**	.032
RE → anger	.052**	.025
RE → advocacy intentions	.187***	.019
*Indirect*		
Threat → fear → advocacy intentions	.032***	.013
Threat → anger → advocacy intentions	.015*	.011
SE → fear → advocacy intentions	-.003	.002
SE → anger → advocacy intentions	.003*	.001
RE → fear → advocacy intentions	.004*	.002
RE → anger → advocacy intentions	.002	.001
*Total*		
Threat	.092***	.027
SE	.639***	.030
RE	.193***	.019

Note.

*p < .05

**p < .01

***p < .001.

All path coefficients in the final model were statistically significant (*p* < .05) (Fig 2). Self-efficacy was negatively related to fear (*β* = -.05, *p* = .011; H4 supported), positively related to anger (*β* = .08, *p* < .001; RQ2), and directly predicted the intention to engage in advocacy (*β* = .64, *p* < .001; H1 supported). Collective response efficacy was positively related to both fear (*β* = .05, *p* = .004; H5 not supported) and anger (*β* = .05, *p* = .004; RQ3) and directly predicted the intention to engage in advocacy (*β* = .19, *p* < .001; H2 supported). Perceived threat was positively related to both fear (*β* = .45, *p* < .001; H6 supported) and anger (*β* = .42, *p* < .001; H8 supported) and directly predicted the intention to engage in advocacy (*β* = .04, *p* = .013; H3 supported). Fear (*β* = .07, *p* < .001; H7 supported) and anger (*β* = .04, *p* = .013; H9 supported) both directly predicted the intention to engage in advocacy. Race (*β* = -.02, *p* = .290), education (*β* = -.03, *p* = .100), and age (*β* = -.03, *p* = .057) did not directly predict fear, while gender did (*β* = .10, *p* < .001). Gender (*β* = -.001, *p* = .963), race (*β* = -.01, *p* = .430), and education (*β* = .01, *p* = .609) did not directly predict anger, while age did (*β* = .08, *p* < .001). Age (*β* = .02, *p* = .111), race (*β* = -.02, *p* = .081), and education (*β* = -.006, *p* = .651) did not directly predict intentions to engage in advocacy, while gender did (*β* = .03, *p* = .034).

These results confirm all our hypotheses except for H5, as collective response efficacy had a positive relationship with fear rather than the hypothesized negative relationship. Further, the results give insight into those relationships for which we could not hypothesize a directional relationship, demonstrating that self- and collective response efficacy have positive relationships with anger (RQ1 and RQ2).

## Discussion

In 2017, the March for Science drew international attention and tens of thousands gathered to show their support for science and its role in society. Our analysis of survey data collected from people on the March’s listserv confirmed the majority of our hypotheses about predictors of intentions to engage in further advocacy to defend science. Specifically, self- and collective response efficacy, perceived threat to science, anger, and fear predicted intentions to engage in further advocacy.

Our findings advance theoretical understandings of what factors lead to the intent to engage in advocacy to defend science. Although the EPPM has primarily been used to understand how people respond to concrete personal health risks, our study suggests a modified version of the model (i.e., the addition of negative emotions) can be successfully extended and applied in the context of advocacy intentions to defend science; this aligns with previous extensions of the EPPM [22,23]. More specifically, our model reinforced that perceptions of threats, feelings of efficacy (self- and response), and emotions (fear and anger) help explain intentions to engage in advocacy to defend science. Further, in line with prior research [21–23,27,28], we found that self- and collective response efficacy are positively related to the intention to engage in advocacy, with self-efficacy having the strongest relationship in the model. This reaffirms the well-established importance of efficacy to advocacy intentions as well as behavioral intentions broadly.

The relationships between self- and collective response efficacy and advocacy intentions have important implications for efforts to increase advocacy engagement. Increasing individuals’ self-efficacy about their ability to engage in advocacy has the greatest potential to help increase peoples’ intentions to engage in advocacy to defend science. Similarly, increasing perceptions that those actions will make a difference may also enhance intentions to engage in advocacy. Communication efforts to increase advocacy intentions should therefore make appeals to self- and collective response efficacy by demonstrating that others are engaging in advocacy, providing information about the different ways to engage in advocacy, training people in how to engage in advocacy to increase their own sense of self-efficacy, modeling these behaviors, and showing people that their engagement in advocacy can help achieve the goals of the action. Additionally, at least one survey found that many individuals say they do not engage in climate advocacy simply because they have never been asked to do so [49], which suggests that new methods to identify and reach those already willing to engage could prove beneficial.

The March was organized directly in response to perceived threats to science and evidence-based decision-making, and members of their listserv may therefore perceive similar threats. Here, we found that perceived threat was also positively related to the intention to engage in advocacy to defend science. Therefore, highlighting threats to science may be a strategy to increase advocacy intentions. However, this strategy should be exercised with caution because previous research has found that when threats to science are framed as a “war on science” and are perceived as overly aggressive, it can cause ideological polarization such that liberals view scientists as more credible and conservatives view them as less credible [43]. Future research should test whether it is possible to talk about threats to science in a way that encourages political participation while minimizing unintended polarization in attitudes toward scientists.

Fear and anger were related to all variables in the model. Though small, there were positive relationships between fear and anger and the intention to engage in advocacy to defend science. These findings reinforce the relationships put forth by the SIMCA model, in which negative emotions (primarily anger resulting from perceived injustices) lead to collective action [21]. Additionally, though in the context of health behavior intentions, a 2015 meta-analysis demonstrated that fear appeals were effective at increasing behavioral intentions [50]. Another study found that fear appeals were positively related to signing petitions [51]. Therefore, the use of fear appeals in driving intentions (and behaviors) shows promise in other contexts, but their continued exploration in the context of advocacy is needed. Our findings suggest that making direct, emotional appeals to fear and anger may further promote intentions to engage in advocacy. However, our study does not test the boundaries of these emotions, so caution should be taken when crafting messaging that appeals to fear and anger to avoid having debilitating impacts on the target audience. Future research should investigate this further.

In addition to making direct appeals, appeals to efficacy and perceived threat may also influence feelings of fear and anger. Perceived threat had the strongest relationship to fear and anger—further exemplifying the relationship put forth by the EPPM and to a lesser extent the role of perceived injustices in SIMCA. These relationships are important to consider, as we demonstrate here that they may be motivating the intent to engage in advocacy rather than demotivating it. Again, the March serves as an example of how threat perceptions and emotional responses may lead to mass mobilization.

The effects of collective response efficacy on fear and anger are comparable in magnitude to those of self-efficacy; however, collective response efficacy was positively related to both fear and anger, while self-efficacy was positively related to anger and negatively related to fear. The reason for the different relational directions between fear and self- and collective response efficacy is less clear. Fear occurs when threats seem out of one’s control [22]. Our measurement and conceptualization of response-efficacy focused specifically on collective response efficacy (i.e., the perception of the effectiveness of the advocacy measured if “many other people who share your views do” those actions). This highlights the necessity of advocacy and the uncertainty surrounding whether other like-minded individuals will engage in the actions, therefore lowering how much control an individual may perceive over the situation and leading to higher feelings of fear. Further, collective response efficacy, particularly as we measured it, may allude to a social norm surrounding the action, which can also play a role in motivating people’s intentions to and actual engagement [52,53]. On the other hand, self-efficacy may make an individual feel as though they have the ability to engage in advocacy, creating a larger sense of control and therefore decreasing the amount of fear they feel. Future research should continue to examine how different types of efficacy relate to different emotions.

Research on political opportunity structures has extensively examined the role of perceived threat in the form of repression; this extreme type of threat has been theorized to depress action [54–56]. This literature’s focus on the broader structural and environmental factors that function to motivate or depress action (as opposed to the strictly psychological factors we examine in the current study) may be a potentially fruitful lens for future research on advocacy to defend science.

Taken together, our results provide insights into what drives people’s intentions to engage in advocacy to protect science, like the March. This builds on prior research into the biographical consequences of activism which found that people who engage in high-risk or time-consuming actions (such as attending rallies) are more likely to remain engaged in advocacy behavior over time compared to people who did not engage in such actions [57,58]. Therefore, our findings may be especially important when considering the long-term goals of demonstrations like the March, as furthering understandings of how to increase people’s intentions to engage in advocacy and their actual engagement is crucial to growing and sustaining social movements in the defense of science.

## Limitations

There are several limitations associated with this study. First, because our data are cross-sectional, any causal inferences must be interpreted with caution. We also only looked at intentions to engage in advocacy, rather than the actual behavior, and there is evidence that intentions do not necessarily predict behaviors [59]. Future work should seek to investigate advocacy behaviors directly, as this would be a stronger measure of the role the constructs in this study play in predicting advocacy behaviors. Further, we only examined intentions to engage in certain types of advocacy and therefore we cannot make claims about the relationships in the variables in this study with other forms of advocacy. Additionally, there are likely other predictors that may influence intentions to engage in advocacy to defend science beyond those that are included in our model, such as other emotional responses, media influences, issue involvement, attitudes towards advocacy, and perceived barriers to advocacy [60–62]. In particular, the lack of an identity measure is an additional limitation of this study, as this is a core component of SIMCA and therefore is likely important to explore in the context of advocacy for science. We also did not test the interaction between perceived threat and efficacy that is implicit in the EPPM. Therefore, investigating other predictors and interactions are avenues for future research.

Our model did not reach desired levels of model fit due to our choice to opt for parsimony within the model. Future studies should continue to examine how to improve the model proposed here. It is also important to note that our results are not generalizable to the broader public or to those who attended the March, as only members of the March for Science listserv were surveyed. The findings presented give us insights into the audience that felt compelled to join the March for Science listserv as of Spring 2017 and care about issues surrounding the role of science in society. Lastly, the sample was predominantly White, female, highly educated, and likely skewed liberal, although political ideology was not measured. This underscores the need for more research on how to broaden the diversity of this social movement for science. Greater educational, ethnic, and racial diversity would help to ensure that the policy victories of this movement benefit historically marginalized communities, and greater ideological/partisan diversity would help depolarize debates around these issues.

## Conclusion

The 2017 March for Science was a substantial show of support in defense of science and provided a unique opportunity to investigate how those interested in the March may engage in further advocacy to defend science. To promote and protect the role of science in society and to continue achieving the level of success experienced by the March, the sustained activation of both scientists and nonscientists to engage in advocacy is vital. Our work adds to understandings of how to achieve this goal by providing theoretical and practical insights into how to increase the intentions of individuals to engage in advocacy to defend science and opens new doors for future research. However, understanding of the drivers of science advocacy engagement alone is not enough to preserve the role of science in society. A multifaceted approach is needed to implement strategies to encourage individuals to act.

## Supporting information

S1 File Means, standard deviations, and correlations with confidence intervals. (DOCX)
