# Supplementary material for: What motivates people to defend science: Evidence from the 2017 March for science
Source: PLoS One. 2023 Nov 16;18(11):e0290222. doi: 10.1371/journal.pone.0290222 (PMC10653542; doi:10.1371/journal.pone.0290222)
Supplement: S1 File — (DOCX) [file pone.0290222.s001.docx]

S1 Table

*Means, standard deviations, and correlations with confidence intervals*

| Variable | *M* | *SD* | 1 | 2 | 3 | 4 | 5 | 6 | 7 | 8 | 9 | 10 | 11 | 12 | 13 | 14 | 15 | 16 | 17 | 18 | 19 | 20 | 21 |
| --- | --- | --- | --- | --- | --- | --- | --- | --- | --- | --- | --- | --- | --- | --- | --- | --- | --- | --- | --- | --- | --- | --- | --- |
|  |  |  |  |  |  |  |  |  |  |  |  |  |  |  |  |  |  |  |  |  |  |  |  |
| 1. PT1 | 4.52 | 0.76 |  |  |  |  |  |  |  |  |  |  |  |  |  |  |  |  |  |  |  |  |  |
|  |  |  |  |  |  |  |  |  |  |  |  |  |  |  |  |  |  |  |  |  |  |  |  |
| 2. PT2 | 4.12 | 0.94 | .54** |  |  |  |  |  |  |  |  |  |  |  |  |  |  |  |  |  |  |  |  |
|  |  |  | [.51, .56] |  |  |  |  |  |  |  |  |  |  |  |  |  |  |  |  |  |  |  |  |
|  |  |  |  |  |  |  |  |  |  |  |  |  |  |  |  |  |  |  |  |  |  |  |  |
| 3. PT3 | 3.99 | 1.08 | .46** | .74** |  |  |  |  |  |  |  |  |  |  |  |  |  |  |  |  |  |  |  |
|  |  |  | [.44, .48] | [.73, .75] |  |  |  |  |  |  |  |  |  |  |  |  |  |  |  |  |  |  |  |
|  |  |  |  |  |  |  |  |  |  |  |  |  |  |  |  |  |  |  |  |  |  |  |  |
| 4. PT4 | 4.28 | 0.94 | .47** | .59** | .61** |  |  |  |  |  |  |  |  |  |  |  |  |  |  |  |  |  |  |
|  |  |  | [.45, .50] | [.57, .61] | [.60, .63] |  |  |  |  |  |  |  |  |  |  |  |  |  |  |  |  |  |  |
|  |  |  |  |  |  |  |  |  |  |  |  |  |  |  |  |  |  |  |  |  |  |  |  |
| 5. PT5 | 4.50 | 0.77 | .46** | .52** | .44** | .49** |  |  |  |  |  |  |  |  |  |  |  |  |  |  |  |  |  |
|  |  |  | [.43, .48] | [.49, .54] | [.42, .47] | [.47, .51] |  |  |  |  |  |  |  |  |  |  |  |  |  |  |  |  |  |
|  |  |  |  |  |  |  |  |  |  |  |  |  |  |  |  |  |  |  |  |  |  |  |  |
| 6. PT6 | 4.13 | 1.01 | .42** | .53** | .48** | .49** | .53** |  |  |  |  |  |  |  |  |  |  |  |  |  |  |  |  |
|  |  |  | [.40, .44] | [.51, .55] | [.46, .50] | [.47, .51] | [.51, .56] |  |  |  |  |  |  |  |  |  |  |  |  |  |  |  |  |
|  |  |  |  |  |  |  |  |  |  |  |  |  |  |  |  |  |  |  |  |  |  |  |  |
| 7. PT7 | 4.19 | 1.00 | .43** | .52** | .50** | .47** | .48** | .58** |  |  |  |  |  |  |  |  |  |  |  |  |  |  |  |
|  |  |  | [.40, .45] | [.50, .54] | [.48, .52] | [.45, .49] | [.46, .50] | [.56, .59] |  |  |  |  |  |  |  |  |  |  |  |  |  |  |  |
|  |  |  |  |  |  |  |  |  |  |  |  |  |  |  |  |  |  |  |  |  |  |  |  |
| 8. Fear | 3.60 | 1.20 | .26** | .35** | .33** | .30** | .29** | .32** | .31** |  |  |  |  |  |  |  |  |  |  |  |  |  |  |
|  |  |  | [.23, .29] | [.32, .37] | [.30, .35] | [.28, .33] | [.26, .32] | [.29, .34] | [.29, .34] |  |  |  |  |  |  |  |  |  |  |  |  |  |  |
|  |  |  |  |  |  |  |  |  |  |  |  |  |  |  |  |  |  |  |  |  |  |  |  |
| 9. Anger | 4.41 | 0.90 | .31** | .32** | .29** | .27** | .29** | .30** | .30** | .39** |  |  |  |  |  |  |  |  |  |  |  |  |  |
|  |  |  | [.28, .34] | [.30, .35] | [.26, .32] | [.25, .30] | [.27, .32] | [.27, .33] | [.27, .33] | [.37, .42] |  |  |  |  |  |  |  |  |  |  |  |  |  |
|  |  |  |  |  |  |  |  |  |  |  |  |  |  |  |  |  |  |  |  |  |  |  |  |
| 10. SE1 | 3.42 | 1.24 | .10** | .09** | .08** | .11** | .09** | .10** | .09** | .04* | .12** |  |  |  |  |  |  |  |  |  |  |  |  |
|  |  |  | [.07, .13] | [.06, .12] | [.05, .11] | [.08, .14] | [.06, .12] | [.07, .13] | [.06, .12] | [.01, .07] | [.09, .15] |  |  |  |  |  |  |  |  |  |  |  |  |
|  |  |  |  |  |  |  |  |  |  |  |  |  |  |  |  |  |  |  |  |  |  |  |  |
| 11. SE2 | 3.36 | 1.26 | .09** | .05** | .03 | .07** | .05** | .05** | .05** | -.03 | .06** | .32** |  |  |  |  |  |  |  |  |  |  |  |
|  |  |  | [.06, .12] | [.02, .08] | [-.00, .06] | [.04, .10] | [.02, .08] | [.02, .08] | [.02, .08] | [-.06, .00] | [.03, .09] | [.29, .34] |  |  |  |  |  |  |  |  |  |  |  |
|  |  |  |  |  |  |  |  |  |  |  |  |  |  |  |  |  |  |  |  |  |  |  |  |
| 12. SE3 | 3.91 | 1.01 | .13** | .13** | .12** | .14** | .14** | .15** | .13** | .12** | .18** | .44** | .35** |  |  |  |  |  |  |  |  |  |  |
|  |  |  | [.10, .16] | [.10, .16] | [.09, .15] | [.11, .17] | [.11, .17] | [.12, .18] | [.10, .16] | [.09, .15] | [.15, .20] | [.41, .46] | [.33, .38] |  |  |  |  |  |  |  |  |  |  |
|  |  |  |  |  |  |  |  |  |  |  |  |  |  |  |  |  |  |  |  |  |  |  |  |
| 13. SE4 | 3.43 | 1.34 | .09** | .11** | .13** | .12** | .06** | .10** | .14** | .04** | .09** | .29** | .14** | .36** |  |  |  |  |  |  |  |  |  |
|  |  |  | [.06, .12] | [.08, .14] | [.10, .15] | [.09, .15] | [.03, .09] | [.07, .13] | [.11, .17] | [.01, .07] | [.06, .12] | [.26, .32] | [.11, .16] | [.34, .39] |  |  |  |  |  |  |  |  |  |
|  |  |  |  |  |  |  |  |  |  |  |  |  |  |  |  |  |  |  |  |  |  |  |  |
| 14. SE5 | 4.13 | 0.95 | .10** | .08** | .07** | .07** | .08** | .09** | .10** | .02 | .11** | .33** | .21** | .44** | .51** |  |  |  |  |  |  |  |  |
|  |  |  | [.07, .13] | [.05, .11] | [.04, .10] | [.04, .10] | [.05, .11] | [.06, .12] | [.07, .13] | [-.01, .05] | [.08, .14] | [.30, .36] | [.18, .24] | [.41, .46] | [.49, .54] |  |  |  |  |  |  |  |  |
|  |  |  |  |  |  |  |  |  |  |  |  |  |  |  |  |  |  |  |  |  |  |  |  |
| 15. SE6 | 2.65 | 1.28 | .03* | .06** | .04** | .03* | .02 | .07** | .07** | -.00 | .08** | .35** | .14** | .29** | .37** | .36** |  |  |  |  |  |  |  |
|  |  |  | [.00, .06] | [.03, .09] | [.01, .07] | [.00, .06] | [-.01, .05] | [.04, .10] | [.04, .10] | [-.03, .03] | [.05, .11] | [.32, .37] | [.11, .17] | [.26, .32] | [.35, .40] | [.34, .39] |  |  |  |  |  |  |  |
|  |  |  |  |  |  |  |  |  |  |  |  |  |  |  |  |  |  |  |  |  |  |  |  |
| 16. RE1 | 3.36 | 1.09 | .04** | .04** | .05** | .06** | .04* | .05** | .04** | .06** | .06** | .23** | .13** | .20** | .08** | .10** | .11** |  |  |  |  |  |  |
|  |  |  | [.01, .07] | [.01, .07] | [.02, .08] | [.03, .09] | [.01, .07] | [.02, .08] | [.01, .07] | [.03, .09] | [.03, .09] | [.20, .26] | [.10, .16] | [.17, .23] | [.05, .11] | [.07, .13] | [.08, .14] |  |  |  |  |  |  |
|  |  |  |  |  |  |  |  |  |  |  |  |  |  |  |  |  |  |  |  |  |  |  |  |
| 17. RE2 | 3.42 | 0.96 | .10** | .12** | .12** | .13** | .11** | .11** | .12** | .12** | .11** | .18** | .29** | .23** | .11** | .13** | .09** | .55** |  |  |  |  |  |
|  |  |  | [.07, .13] | [.09, .15] | [.09, .15] | [.10, .16] | [.08, .14] | [.08, .14] | [.09, .15] | [.10, .15] | [.08, .14] | [.15, .21] | [.26, .32] | [.20, .26] | [.08, .14] | [.10, .16] | [.06, .12] | [.53, .57] |  |  |  |  |  |
|  |  |  |  |  |  |  |  |  |  |  |  |  |  |  |  |  |  |  |  |  |  |  |  |
| 18. RE3 | 3.11 | 1.00 | .06** | .13** | .12** | .12** | .10** | .11** | .09** | .14** | .15** | .14** | .08** | .32** | .12** | .14** | .15** | .52** | .52** |  |  |  |  |
|  |  |  | [.03, .09] | [.10, .16] | [.10, .15] | [.09, .15] | [.07, .13] | [.08, .14] | [.06, .12] | [.11, .17] | [.12, .18] | [.11, .16] | [.05, .11] | [.30, .35] | [.09, .15] | [.11, .17] | [.12, .18] | [.50, .54] | [.49, .54] |  |  |  |  |
|  |  |  |  |  |  |  |  |  |  |  |  |  |  |  |  |  |  |  |  |  |  |  |  |
| 19. RE4 | 2.82 | 1.12 | .04* | .10** | .10** | .09** | .06** | .08** | .07** | .09** | .12** | .08** | .04** | .19** | .24** | .18** | .20** | .40** | .39** | .60** |  |  |  |
|  |  |  | [.01, .07] | [.07, .13] | [.07, .13] | [.06, .12] | [.03, .09] | [.05, .11] | [.04, .10] | [.06, .12] | [.09, .15] | [.05, .10] | [.01, .07] | [.16, .21] | [.21, .26] | [.15, .21] | [.17, .23] | [.37, .42] | [.37, .42] | [.58, .62] |  |  |  |
|  |  |  |  |  |  |  |  |  |  |  |  |  |  |  |  |  |  |  |  |  |  |  |  |
| 20. RE5 | 3.12 | 1.11 | .03 | .08** | .08** | .07** | .06** | .07** | .09** | .07** | .10** | .09** | .05** | .15** | .15** | .22** | .17** | .37** | .38** | .55** | .68** |  |  |
|  |  |  | [-.00, .06] | [.05, .11] | [.05, .11] | [.04, .10] | [.03, .09] | [.04, .10] | [.06, .12] | [.04, .10] | [.07, .13] | [.06, .12] | [.02, .08] | [.13, .18] | [.12, .18] | [.19, .25] | [.14, .20] | [.34, .39] | [.36, .41] | [.53, .57] | [.66, .70] |  |  |
|  |  |  |  |  |  |  |  |  |  |  |  |  |  |  |  |  |  |  |  |  |  |  |  |
| 21. RE6 | 3.23 | 1.02 | .03 | .05** | .06** | .04** | .03 | .06** | .08** | .08** | .08** | .12** | .06** | .18** | .14** | .18** | .25** | .48** | .43** | .53** | .59** | .56** |  |
|  |  |  | [-.00, .06] | [.02, .08] | [.03, .09] | [.01, .07] | [-.00, .06] | [.03, .09] | [.05, .11] | [.05, .11] | [.05, .11] | [.09, .15] | [.03, .09] | [.15, .21] | [.11, .17] | [.15, .21] | [.22, .28] | [.46, .50] | [.40, .45] | [.50, .55] | [.57, .61] | [.54, .58] |  |
|  |  |  |  |  |  |  |  |  |  |  |  |  |  |  |  |  |  |  |  |  |  |  |  |
| 22. advocacy | 3.36 | 0.78 | .18** | .19** | .19** | .19** | .14** | .20** | .21** | .18** | .23** | .42** | .13** | .40** | .53** | .36** | .45** | .23** | .23** | .32** | .34** | .28** | .30** |
|  |  |  | [.15, .21] | [.16, .22] | [.17, .22] | [.16, .22] | [.11, .17] | [.17, .23] | [.18, .24] | [.15, .21] | [.20, .26] | [.39, .44] | [.10, .16] | [.38, .43] | [.51, .56] | [.33, .39] | [.43, .48] | [.20, .26] | [.20, .26] | [.29, .35] | [.31, .36] | [.25, .31] | [.27, .32] |
|  |  |  |  |  |  |  |  |  |  |  |  |  |  |  |  |  |  |  |  |  |  |  |  |

*Note.* *M* and *SD* are used to represent mean and standard deviation, respectively. Values in square brackets indicate the 95% confidence interval for each correlation. The confidence interval is a plausible range of population correlations that could have caused the sample correlation (Cumming, 2014). * indicates *p* < .05. ** indicates *p* < .01.
